# Chemical Composition and *In Vitro* Activity of Plant Extracts from *Ferula communis* and *Dittrichia viscosa* against Postharvest Fungi

**DOI:** 10.3390/molecules16032609

**Published:** 2011-03-22

**Authors:** Erjon Mamoci, Ivana Cavoski, Vito Simeone, Donato Mondelli, Lina Al-Bitar, Pierluigi Caboni

**Affiliations:** 1 Dipartimento di Science per l'Ambiente, Università degli Studi di Napoli "Parthenope" Centro Direzionale, Isola C4, 80143 Napoli, Italy; 2 Istituto Agronomico Mediterraneo di Bari,Via Ceglie 9, 70010 Valenzano, Italy; 3 Dipartimento di Biologia e Chimica Agro-Forestale ed Ambientale, Università degli Studi di Bari, Via Amendola 165/A, 70126 Bari, Italy; 4 Dipartimento di Tossicologia, Università degli Studi di Cagliari, Via Ospedale 72, 09124 Cagliari, Italy

**Keywords:** plant extracts, sesquiterpenes, coumarin, postharvest fungi

## Abstract

*F. communis* and *D. viscosa* are perennial Mediterranean weeds that have been used for different therapeutic purposes in traditional pharmacopeia. Plant extracts were obtained from air dried *D. viscosa* young shoots (DvA) and *F. communis* aerial part (FcA) and roots (FcR) with *n*-hexane. The chemical compositions of the extracts were analyzed by HPLC-DAD, LC-MS (ESI) and LC-Q-TOF techniques. Two sesquiterpene lactones (inuviscolide, tomentosin) and three sesquiterpene acids (costic acid, hydroxycostic acid, ilicic acid) were identified from the *D. viscosa* extract, while in *F. communis* extracts three daucane sesquiterpenes (acetoxyferutinin, oxojaeskeanadioyl anisate, fertidin) and one coumarin (ferulenol) derivates were found. Biological activities of plant extracts were studied in *in vitro* experiments on the colonies and conidia of *Botryotinia fuckeliana, Penicillium digitatum, P. expansum, Monilinia laxa, M. fructigena* and *Aspergillus spp.* Extracts showed varying degree of antifungal activities on colony growth and conidia germination*.* The extract from FcA showed the least effect, while DvA extract had the strongest fungitoxic effects*.* FcRextract presented a fungitoxic effect on the colony growth, but it was not able to inhibit the conidia germination. These distinctions can be attributed to the differences in chemical composition of plant extracts.

## 1. Introduction

Decay diseases caused by fungi create great postharvest losses of fruits and vegetables. Due to their attack they make the crop unfit for consumption and additionally some postharvest pathogens are able to produce mycotoxins [1,2]. Synthetic fungicides are the main measure used in the management of postharvest decay [3]. Pesticide residues [4] and development of resistant strains of fungi from the continuous application [5,6] are the major constraints to their use. Plant derived compounds are generally assumed to be more acceptable and less harmful than synthetic ones. These compounds possess a high potential for pest management since most of them are not phytotoxic, easily biodegradable and sometimes stimulatory to the host metabolism [7]. Therefore, exploitation of the new alternatives to synthetic fungicides should be considered. 

*Ferula communis* (L.) and *Dittrichia viscosa* (L.) W. Greuter (syn. *Inula viscosa* (L.) Aiton., *Cupularia viscosa* G. et G.) are perennial weeds, native to the Mediterranean basin. In traditional pharmacopeia, several species of *Ferula* genus and *D. viscosa* have been used for different therapeutic purposes [8,9,10].

*F. communis* belongs to the family *Umbelliferae* and different chemical investigations have reported the presence of coumarinosesquiterpenes [11], daucane esters [8,12] and phenylpropanoid compounds [13] in fruits and roots. There are two distinguishable chemotypes of *F. communis:* i) the ‘nonpoisonous’ chemotype, containing as main constituents the daucane esters; and ii) a ‘poisonous’ chemotype containing prenylated coumarins, but missing the daucanes [14]. Prenylcoumarin compounds from *F. communis* (L.) were thought to be responsible for the toxic effects on sheep, goats, cattle and horses [11]. Recently the presence of the toxic prenylcoumarin ferulenol was reported in the ‘non-poisonous’ chemotype of *F. communis* [14,15]. Al-Yahya *et al*. [16] isolated three antibacterial sesquiterpenes 14-(*o*-hydroxycinnamoyloxy)-dauc-4,8-diene, ferulenol and ferchromone from roots of *F. communis*. Another two daucane esters, 2α-acetyl-6α-(benzoyl)-jaeschkenadiol and 2α-acetyl-6α-(*p*-anisoyl)- jaeschkenadiol [17], and two coumarine sesquiterpenes, 2-nor-1,2-secoferulenol [18] and feselol [13], were isolated from roots, but their activities were not studied. Ferulenol isolated from the roots of *F. communis* and its derivates have shown good antimycobacterial activity [19,20], but there are no data that reports their activity on plant pathogenic fungi.

*D. viscosa* belongs to the family *Compositae* and different chemical investigations have reported the presence of flavonoids [21], triterpenoids [22,23], sesquiterpene lactones and acids [24,25,26]. The biological activity of this plant has been subject of different investigations. Formulated leaf extracts of *D. viscosa* were tested for their nematicidial activity in field trials, where only a slight effect was obtained, even though the formulated extract was effective in pot experiments [27]. *D. viscosa* has also been subject of investigation against insects [28] and mites [29]. A sesquiterpene lactone, tomentosin, has been isolated and identified from *D. viscosa* and it was shown to be active under *in vitro* conditions against the dermatophytes *Microsporum canis*, *M. gypseum* and *Trichophyton mentagrophytes* [30]. The same authors observed a greater antifungal activity of leaf extracts, due to the high levels of the sesquiterpene carboxyeudesmadiene, against dermatophytes and *Candida* spp. [31]. *D. viscosa* extract causes a decline in chitin content, a very important constituent of fungal cell wall, which probably explains the antimycotic activity of the plant extract against dermatophytes. The extract caused dramatic changes in the hyphae and spore morphology due to severe damage in the fungal cell coat [32,33]. Qasem *et al.* [34] reported that, *D. viscosa* incorporated as shoot dried material or crude water extract in a culture medium, showed antifungal effects on the mycelial growth of *Fusarium oxysporum* f.sp. *lycopersici*. Abou-Jawdah *et al*. [35] found out that *D. viscosa* has high activity against spore germination, but only moderate activity against mycelial growth of different fungi. Under *in vivo* conditions preventive sprays of *D. viscosa* on squash and cucumber seedlings gave efficient protection against *Botrytis cinerea* and *Sphaerotheca cucurbitae*, but failed to control green mold of citrus fruits caused by *Penicillium* sp. [35]. Extracts of *D. viscosa* made with organic solvents were effective controlling downy mildew in grape [36] and late blight in potato and tomato, downy mildew in cucumber, powdery mildew in wheat and rust in sunflower, under controlled conditions [37]. Cohen *et al*. [38] using an emulsified concentrate formulation of the oily paste extracts provided very good control against downy mildew of grapes caused by *Plasmopara viticola.* The major inhibitory compounds were identified as tomentosin and costic acid [38]. Costic acid has also been sustained to represent one of the principle nematicidal ingredients of *D. viscosa* against *Meloidogyne* sp. [39]. Although the antifungal activities of *D. viscosa* have been studied its possible application in postharvest fungi control remains unexplored.

The aim of this research was to test the *in vitro* antifungal activity of plant extracts from *F. communis* and *D. viscosa* against postharvest fungi of fruits where limited or inexistent information was available and to characterize plant extracts by HPLC-DAD, LC-MS (ESI) and LC-Q-TOF analysis.

## 2. Results and Discussion

### 2.1. Extracted Yield

Extracted yields obtained from air dried plant parts of *D. viscosa* young shoots (DvA), *F. communis* aerial part (FcA) and root (FcR) are presented in Table 1. 

**Table 1:** Plant species, phenological phase, plant extract abbreviation and extracted yields from plant material (mean values followed by ** are significantly different at P < 0.01 according to Duncan’s multiple range test).

Plant species	Phenological phase	Plant extract abbreviation	Extracted yield (g/kg dw)
*D. viscosa*	Leaf-rosette	DvA	76.4
*F. communis*	Flowering		
roots		FcR	79.5
aerial part		FcA	25.1**

The highest yields of 79.5 and 76.4 g/kg were obtained from FcR and DvA, respectively, while the yield from FcA with 25.1 g/kg, was much lower. These differences are attributed to the fact that plant secondary metabolites often accumulate in specific plant parts [40] and their quantity is related to the plant’s phenological stage [41].

### 2.2. Chemical Composition

**Figure 1:**
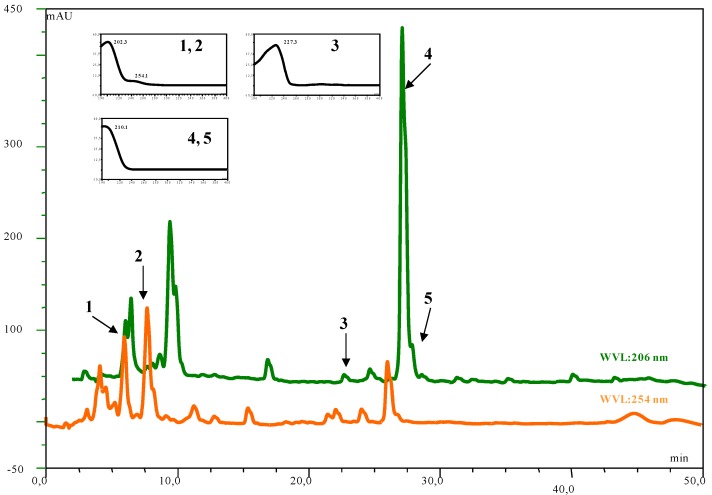
UV spectra at 206 and 254 nm of DvA with UV spectra of **1)** inuviscolide, **2)** tomentosin, **3)** ilicic acid, **4)** costic acid, **5)** 3*α*-hydroxy costic acid.

**Figure 2:**
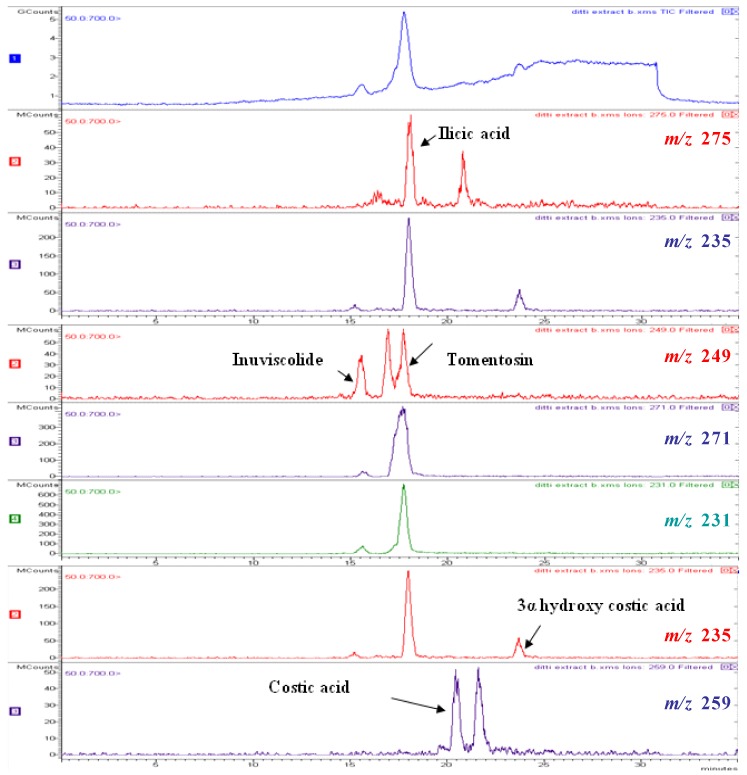
ESI total ion chromatogram of of DvA extract and selected ion monitoring for each compound.

The HPLC-DAD and LC-MS (ESI) analysis of the DvA *n*-hexane soluble fraction allowed us to indentify five compounds (Figure 1 and Figure 2; Table 2). Figure 1 shows the HPLC profiles at 206 and 254 nm of the DvA extract under the optimized conditions of analysis, whereas Figure 2 shows the LC-MS (ESI) profile in positive and negative modes. Table 2 lists the compounds identified with their UV absorption maxima, molecular weights and characteristic adducts. The chromatograms in Figure 1 at 206 nm show the presence of two main peaks tomentosin (2) and costic acid (4). Furthermore, three minor peak compounds **1**, **3**, **5** were found. These peaks could be identified as inuviscolide, ilicic acid and 3α-hydroxycostic acid, respectively.

Peak 1, identified as inuviscolide, presents a λ_max_ at 225 and 254 nm and a positive and negative ESI-MS spectrum comparable with compound **2**, tomentosin. Tomentosin presents a λ_max_ at 254 nm, attributable to its flavonoid chromophore. The positive ESI-MS spectrum of tomentosin exhibits the signals at *m/z* 231 [M-H_2_O+H]^+^, *m/z* 249 [M+H]^+^* m/z* and 271 [M+Na]^+^ and negative ESI-MS ion at *m/z* 247 [M-H]^-^ (Figure 2). Inuviscolide and tomentosin are sesquiterpene lactones with cytotoxic and antibacterial activities isolated from *D. graveolens* [41]. Peak **3**, identified as ilicic acid (eudesmane sesquiterpene derivative) showed a λ_max_ at 227 nm and gives the positive mass signals at *m/z* 275 [M+Na]^+^ and *m/z* 235 [M-H_2_O+H]^+ ^and the negative signal at *m/z* 251 [M-H]^-^ (Figure 2).

**Table 2:** Main signals exhibited in the HPLC-MS^2^ spectra of compounds detected in *D. viscosa* extract and UV Maxima (λ_ max_) detected in HPLC-DAD analysis and proposed attributions.

Compound	Number	λ_ max_	mol. wt.	LC-MS (ESI) *m/z* (amu)
inuviscolide	1	225, 254	248	231 [M-H_2_O+H]^+^; 249 [M+H]^+^; 271 [M +Na]^+^
tomentosin	2	254	248	231 [M-H_2_O+H]^+^; 249 [M+H]^+^; 271 [M +Na]^+^; 247 [M-H]^-^
ilicic acid	3	227	252	275 [M+Na]^+^; 235 [M-H_2_O+H]^+ ^; 251 [M-H]^-^
costic acid	4	210	234	235 [M+H]^+^
3α-hydroxycostic acid	5	210	251	274 [M+Na]^+^

For the high resolution mass spectrometry analysis of pure standards, tomentosin and inuviscolide showed similar spectra with [M+H]^+^, [M+H-H_2_O]^+^ and [M+NH_4_]^+^ adducts. The ppm difference between an observed ion mass/charge and exact ion mass/charge was below 10.03 (Figure 5, Table 3). 

Ilicic acid had been reported as one of the active anti-inflammatory principles of *D. viscosa* [42,43]. Costic acid (peak **4**) presents a λ_max_ at 210 nm in ESI^+^ mode at *m/z* 257 [M+Na]^+^ while its hydroxyl-derivative 3α hydroxycostic acid (peak **5**) gives a signal at *m/z* 252 [M+H]^+^ (Figure 2). *D. viscosa* has been repeatedly studied from the chemical point of view and several sesquiterpene lactones, sesquiterpene acids and flavonoids have been isolated from specimens of diverse origins [14,15,16,17,18,19].

**Table 3:** Q-TOF (ESI^+^) characteristic of inuviscolide, tomentosin, ferunelol. *calculated by Chemdraw Pro 8.0, Cambridge Soft.

Compound	Molecular formula	r. t. (min.)	Log P*	Calculated mass (amu) [M+H]^+^	Measured mass (amu) [M+H]^+^	Error (ppm)
inuviscolide	C_15_H_20_O_3_	4.628	2.15	249.1485	249.1454	10.03418
tomentosin	C_15_H_20_O_3_	4.775	1.56	249.1485	249.1477	3.210936
ferulenol	C_24_H_30_O_3_	10.020	5.68	367.2268	367.2263	1.361556

**Figure 3:**
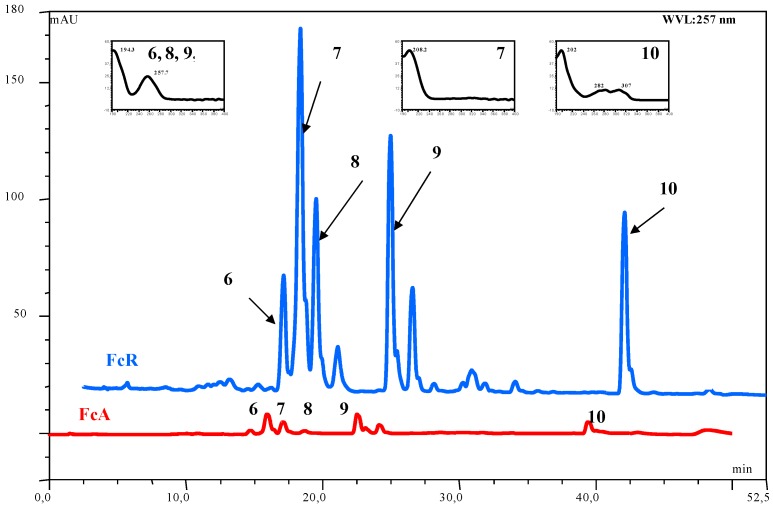
Spectra at 257 of FcA and FcR with UV spectra of **6)** acetoxyferutinin, **7)** unknown, **8)** oxojaeskeanadioyl anisate, **9)** fertidin and **10)** ferulenol.

**Figure 4:**
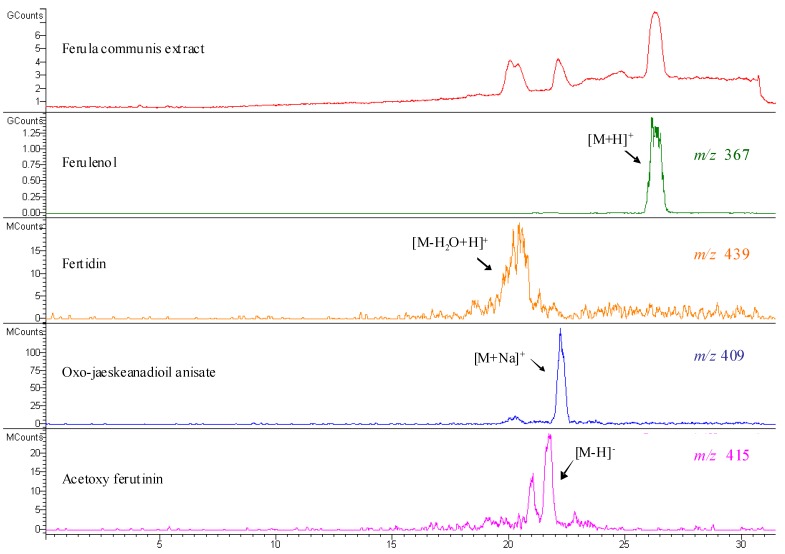
ESI total ion chromatogram of *Ferula communis* extracts and selected ion monitoring for each compound.

**Figure 5:**
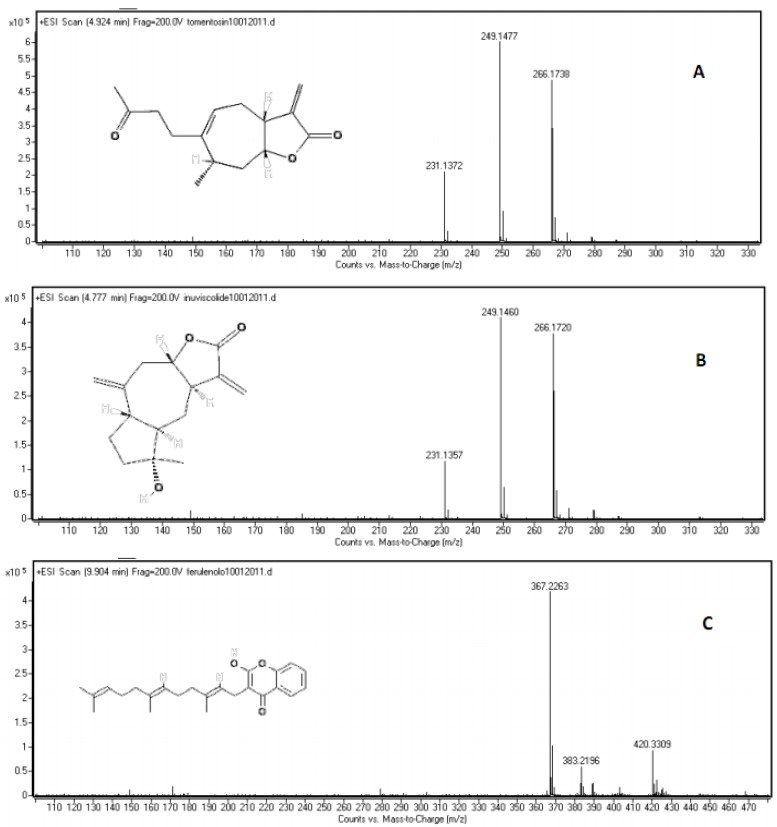
LC-Q-TOF spectra of **A)** tomentosin, **B)** inuviscolide, **C)** ferulenol.

The chromatographic analysis of FcA and FcR *n*-hexane dissolved fraction allowed to identify three daucanes and one coumarin compound (Figure 3 and Figure 4; Table 4). Figure 3 shows the HPLC-UV profile at 257 nm and Figure 4 illustrates the LC-MS (ESI) profile in positive mode of both FcA and FcR extracts. The chromatogram shows the presence of one main peak 7 and four other peaks 6, 8, 9 and 10 (Figure 3). Peaks 6, 8, 9 and 10 could be identified as acetoxy ferutinin, oxo-jaeskeanadiol anisate, fertidin and ferulenol, respectively. The UV spectrum of 7 presents λ_max_ at 208 nm while in positive and negative ESI-MS spectra no spectra were recorded because of that we could not be certain that is lapiferin. Peaks 6, 8 and 9 show UV spectrum of λ_max_ at 257 nm. The ESI negative spectra of compound 6 showed a signal at *m/z* 415 [M-H]^-^, and the ESI positive spectra of 8 and 9 compounds showed the *m/z* 409 [M+Na]^+ ^and *m/z* 439 [M-H_2_O+H]^+^, respectively. Compounds 6, 8 and 9 were, therefore, identified as acetoxy ferutinin, oxo-jaeskeanadioil anisate and fertidin, respectively (Table 4). The UV spectrum of 10 shows λ_max_ 202, 282 and 307 nm and the positive ESI spectra showed [M+H]^+^ signal at *m/z* 367 and [M+Na]^+^ signal at *m/z* 389 while the [M-H]^-^ ion at *m/z* 365 was recorded in the negative ESI-MSspectrum (Figure 4) and was identified as ferulenol. The high resolution mass spectrometry analysis of pure standard showed only [M+H]^+^ and [M+NH_4_]^+^ adducts (Figure 5). The ppm difference between an observed ion mass/charge and exact ion mass/charge was 1.36 (Table 3).

In the FcA extract it is evident lower signals intensity attributed to identified compound in the respect to FcR extract (Figure 4). The differences in the chemical composition between leaves, steam, roots and latex have been reported previously [15]. The components of FcA and FcR extracts were identified by comparing their HPLC retention time, UV absorption maxima and positive and negative mode fragmentation patterns to the corresponding data reported by Arnoldi *et al*. [14].

**Table 4:** Main signals exhibited in the HPLC-MS^2^ spectra of compounds detected in *F. communis* extracts and UV Maxima (λ_ max_) detected in HPLC-DAD analysis and the proposed attributions.

Compound	Number	λ_ max_	mol. wt.	LC-MS (ESI) *m/z* (amu)
acetoxy ferutinin	6	257	416	415 [M-H]^-^
oxojaeskeanadioyl anisate	8	257	386	409 [M+Na]^+^
fertidin	9	257	456	439 [M-H_2_O+H]^+^
ferulenol	10	202, 282, 307	366	367 [M+H]^+^; 365 [M-H]^-^; 389 [M+Na]^+^

The quantity of inuviscolide and tomentosin from *D. viscosa* and ferulenol from *F. communis* extracts expressed as mg/g of extracts are presented in Table 5.

**Table 5:** Content of inuviscolide, tomentosin and ferulenol in plant extracts.

Extract	Compounds			
	inuviscolide	tomentosin	ferulenol	others
	(mg/g extract)			(%)
DvA	42.68	205.80	-	75.15
FcR	-	-	88.40	91.16
FcA	-	-	28.20	97.18

### 2.3. Antifungal Activities

Data of ED_50_s of the extracts on colony growth and conidial germination inhibitions are presented in Table 6.

**Table 6:** ED_50 _(mg/L) and MIC (mg/L, between the brackets) of the extracts on colony growth inhibitions after two (*B. fuckeliana*), six (*P.digitatum*), seven (*M. laxa* and *M. fructigena*), nine (*Aspergillus* spp. and *P. expa*nsum) days and conidial germination inhibitions after 10-12 h of incubations. M: colony growth; C: conidial germination; nd: not determined due to lack of inhibition; ─ not tested.

Extracts	*Aspergillius* spp*.*		*B. fuckeliana*		*P. expansum*		*P. digitatum*		*M. laxa*		*M. fructigena*	
	M	C	M	C	M	C	M	C	M	C	M	C
**DvA**	>400	371	153	192	>400	201	154	83	25	106	29	94
		(nd)	(400)	(400)		(400)	(400)	(400)	(200)	(300)	(200)	(300)
**FcR**	>400	nd	136	>400	>400	nd	>400	>400	202	nd	75	nd
			(400)						(400)		(400)	
**FcA**	>400	─	>400	─	>400	─	>400	─	>400	─	>400	─

DvA was found to inhibit the colony growth of the fungi in a dose dependent manner. The highest antifungal activity of this extract was found on *M. laxa* and *M. fructigena* with ED_50_ 25.4 and 29 mg/L respectively, followed by *B. fuckeliana* (ED_50_ 153 mg/L) and *P. digitatum* (ED_50_ 154 mg/L). The lowest antifungal activity of this extract was found on *Aspergillus* spp. and on *P. expansum* andcalculated ED_50_s were higher than the range of the used concentrations. DvA extract showed inhibition of conidial germination of all tested fungi. The ED_50_s of 371, 192, 201, 83, 106 and 94mg/L were calculated for *Aspergillu*s spp,*B. fuckeliana*, *P. expansum, P. digitatum, M. laxa* and *M. fructigena*, respectively. DvA demonstrated relatively high activity on conidia germination but very low activity on colony growth inhibition of *Aspergillus* spp. and *P. expansum.* These results confirm the results found by Abou-Jawdah *et al*. [35] where extracts of *D. viscosa* showed high activity against spore germination but only moderate activity against mycelial growth. There are different publications that demonstrate the activity of *D. viscosa* extracts against dermatophytes [30,31,32,33] and fungal pathogens [35,45,46] under *in vitro* conditions. However, there are no data that reports activities of *D. viscosa* extract on *M. laxa* and *M. fructigena*, even though in our experimental conditions it showed the highest effects. FcR show very low activity on the colonies of *Aspergillus* spp., *P. expansum* and *P. digitatum* whereas, oncolony growthof *M. fructigena, B. fuckeliana* and *M. laxa* it was found to have a good fungitoxic effect with ED_50_ of 75, 135 and 202 mg/L, respectively. No inhibitory activity of FcR was found on conidial germination in all tested fungi. FcA extract showed neglected inhibitory activity on tested fungi regarding colony growth inhibition under our experimental conditions. Therefore, the activity of this extract was not tested against the conidial germination. MICs values in most cases were of 400 mg/L except for *M. laxa* and *M.fructigena* of 200 and 300 mg/L for mycelia and conidia, respectively. In the case of *Aspergillus* spp. at 400 mg/L only 90% of mycelia growth inhibition was archived and MIC was not determined. 

**Figure 6:**
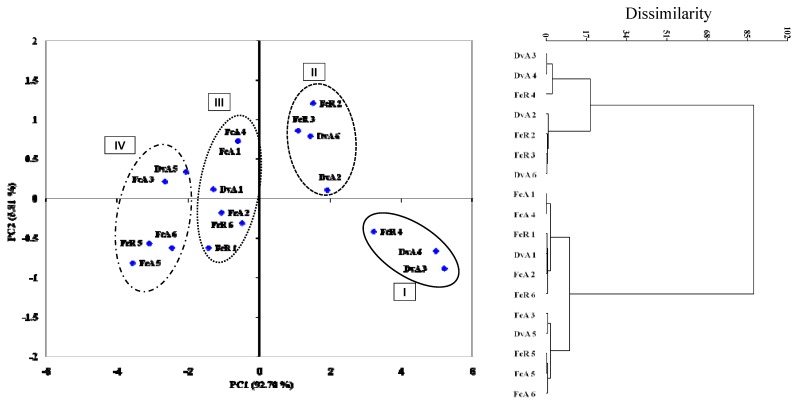
Classification of plant extracts according to colony growth of six postharvest fungi by PCA and cluster analysis. The percentage of total variance explained by each axis in PCA is shown. Numbers from 1 to 6, represent the pathogens *Aspergillus* spp., *B. fuckeliana*, *M. laxa*, *M. fructigena*, *P. expansum* and *P. digitatum*, respectively.

Principal component analysis (PCA) on the colony growth inhibitions and cluster analysis separate extracts into four different groups (Figure 6). Figure 6 shows that the principal components PC1 and PC2 accounted for 98.51% of the variation. Based on PCA and cluster analysis, I group contains DvA 3,4, FcR extracts, II DvA 2, 6, FcR 2,3, III DvA 1, FcA 2,4, FcR 1,6 and IV DvA 5, FcA 3, 5, 6, FcR 5 (Figure 6).

**Figure 7:**
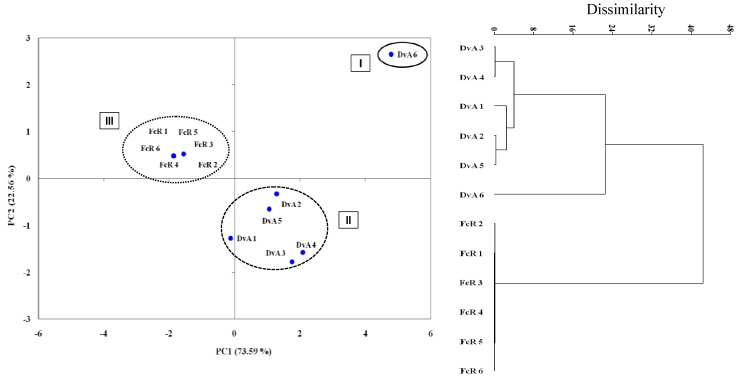
Classification of plant extracts according to conidia germination of six postharvest fungi by PCA and cluster analysis. The percentage of total variance explained by each axis in PCA is shown. Numbers from 1 to 6, represent the pathogens *Aspergillus* spp., *B. fuckeliana*, *M. laxa*, *M. fructigena*, *P. expansum* and *P. digitatum*, respectively*.*

The activity of plant extracts on conidial germination clearly distinguishes three different groups (Figure 7). Each one occupied very different ordinal spaces, indicating that inhibition differed substantially among the treatments. Figure 7 shows that the principal components PC1 and PC2 accounted for 96.15% of the variation. Based on PCA and cluster analysis, there are three different groups: I DvA 6 (main inhibition), II DvA 1 to 5 (intermediate) and III FcR 1 to 6 (minor inhibition) (Figure 7). The effectiveness on colony growth and conidial germination varied among the extracts and tested fungi based on PCA and cluster analysis.

## 3. Experimental

### 3.1. Plant Material and Extraction

Plants were collected from rural areas in Apulia Region, south Italy. *F. communis* (L.) (Giant fennel) was collected during the flowering season and *D. viscosa* (L.) W. Greuter (False yellow head), during the leaf-rosette phenological phase. A voucher specimen was deposited at the Herbarium of the Botany Department, University of Bari. All samples were ground by a blade mill to a fine powder before extraction. Oleoresins were obtained by *n*-hexane extraction in a Soxhlet apparatus. Five grams of samples were extracted with 120 mL *n*-hexane for 6 hours at 68 ºC with 20 min/cycle. At the end of extraction process, solvent in the flask was separated from the oleoresins by evaporation at 40 ºC overnight. The content of oleoresin was corrected according to the dry weight of samples at 105 ºC for 24 hours (till constant weight). Extracts were stored in a freezer at -20 °C until use.

### 3.2. Materials

Sterile plastic Petri dishes used for *in vitro* antifungal trials were purchased from Barloworld Scientific Ltd (Stone, Stafordshire , UK). Organic solvents methanol and acetonitrile (HPLC grade) were purchased from Carlo Erba (Rodano, Milano Italy), whereas Tween 80 and *n*-hexane (99% of purity) were obtained from Sigma-Aldrich (Steinheim, Germany). The filter papers, Whatman No. 1, were purchased from Baker (Mallinckrodt Baker, The Netherlands), potato dextrose agar (PDA), potato dextrose broth (PDB) and glucose from Oxoid LTD (Basingstore, Hampshire, UK). The filter paper (Miracloth) and Thoma counting chamber used for conidia suspension preparation were purchased from Calbiochem (California, USA) and HGB Henneberg-Sander GmbH (Lutzellinden, Germany), respectively. Standards of inuviscolide, tomentosin and ferulenol were kindly provided by Dr. Azucena González-Coloma (Instituto de Ciencias Agrarias, ICA-CSIC, Madrid, Spain).

### 3.3. HPLC-DAD Analysis

The Ultimate 3000 HPLC system (Dionex, Germering, Germany) was equipped with a photodiode array detector, fluorescence detector (FR 2000), Ultimate 3000 low pressure pump pump, Rheodyne (Rheodyne, USA) 20 μL injector loop, Acclaim C18 reverse phase column (150 × 4.6 mm; 3 μm) and Acclaim C18 reverse phase precolumn (10 × 4.6 mm; 5 μm) and a column oven. The HPLC was controlled by and data were elaborated using the Chromeleon Software *vs* 6.8 (Dionex, Germering, Germany). The gradient profile for the separation of *F. communis* and *D. viscosa* extracts was as follows: starting from acetonitrile/water (45:55, v/v) for 5 min, then linear gradient to 15% water in 40 min, maintained for 5 min at acetonitrile/water (85:15, v/v) and equilibrated for 5 min with acetonitrile/water (45:55, v/v) at a flow rate of 1 mL/min. Analyses were performed at UV wavelengths of 197, 254 nm for *F. communis* extracts and 206, 257 nm for *D. viscosa* extract and PDA detection range was 190-400 nm. The external calibrations were provided, and the standard calibration curves were constructed by plotting concentration against peak area. Good linearities were achieved for inuviscolide, tomentosin and ferulenol between 0.01 and 5 mg/L, with a correlation coefficient of 0.9999.

### 3.4. HPLC/ESI-MS/MS Analysis

A Varian tandem mass spectrometer (Palo Alto, CA, USA) consisting of a ProStar 410 autosampler, two ProStar 210 pumps and a 1200 L triple quadrupole mass spectrometer equipped with an electrospray ionization source was used to analyze the plant extracts. Varian MS workstation, version 6.7 software was used for data acquisition and processing. Chromatographic separation was performed on an XDB column (2.1 × 250 mm I.D., particle size 5 *μ*m, Milford, MA). The mobile phase consisted of (A) acetonitrile and (B) bidistilled water with 0.1% of formic acid. Elution started with acetonitrile/water (10:90, v/v) reaching acetonitrile/water (100:0, v/v) in 20 min and hold to 25 min than equilibration time for 10 min till acetonitrile/water (10:90, v/v). The mobile phase, previously degassed with high-purity helium, was pumped at a flow rate of 0.3 mL/min and the injection volume was 10 μL. The electrospray ionization-mass spectrometer was operated in the positive ion mode. The electrospray capillary potential was set to 50 V, while the shield was at 225 V. Nitrogen at 49 mTorr was used as a drying gas for solvent evaporation. The atmospheric pressure ionization (API) housing and drying gas temperatures were kept at 50 and 380 °C, respectively. The scan time was 1 s, and the detector multiplier voltage was set to 1500 V, with an isolation width of *m/z* 1.2 for quadrupole 1 and *m/z* 2.0 for quadrupole 3. ESI mass spectra were acquired modes by scanning over the 50-700 mass range.

### 3.5. HPLC-MS Q-TOF Analysis

The extract was analyzed by reverse phase HPLC on an Agilent 1200 series HPLC system fitted with microchip technology column (Agilent, Zorbax 300 SB-C18 5 μm, 43 mm75 μm). The HPLC conditions were as follows: flow rate, 0.4 μL/min; solvent A, 0.1% formic acid in water; solvent B, methanol; gradient, solvent B 20-100% over 10 min and kept at 100% for 5 min. Then 2 μL of the extract dissolved in methanol-water (80:20, v/v), was analyzed by ESI in positive mode using an Agilent 6520 time-of-flight (TOF) MS. Mass spectral data were acquired in the range *m/z* 100-1000, with an acquisition rate of 1.35 spectra/s, averaging 10,000 transients. The source parameters were adjusted as follows: drying gas temperature 250 C, drying gas flow rate 5 L/min, nebulizer pressure 45 psi, and fragmentor voltage 150 V. Data acquisition and processing were done using Agilent Mass Hunter Workstation Acquisition v. B.02.00 software.

### 3.6. Isolates and Conidial Suspension Preparation

Isolates of *M. laxa, M. fructigena,* *B. fuckeliana, P. digitatum*, *P. expansum* and *Aspergillus* spp. used in these experiments were kindly provided by Prof. Antonio Ippolito from the current collection of the Plant Protection and Applied Microbiology Department, Faculty of Agriculture, University of Bari, Italy. Conidia, obtained from 7-day-old cultures *B*. *fuckeliana,* *P. digitatum*, *P. expansum* *Aspergillus* spp. grown on PDA and *M. laxa* and *M. fructigena* grown on peach infected fruits with 12 h/day exposure to a combination of two daylight (Osram, L36W/20) and 2 near-UV (Osram, L36/73), were collected by scraping the surface of the colonies and fruits, suspended in sterile water containing 0.05% Tween 20 and filtered through Miracloth to remove mycelium fragments. The spore concentrations were adjusted by Thoma counting chamber.

### 3.7. Colony Growth Inhibition Test

The effect of plant extracts postharvest fungi was tested using a food poison technique. Aliquots of plant extracts diluted in methanol were added to the autoclaved culture medium PDA, when the temperature of the medium was 50 ºC, to reach final concentration of 5, 10, 50, 100, 200, 300 and 400 mg/L. Final concentration of methanol didn’t exceed 0.5%. To increase the dispersion of extracts 0.1% of Tween 80 was added. Control in this test was PDA with 0.5% of MeOH and 0.1% of Tween 80. 13 mL of testing and control solutions were added to Petri dishes. All treatments were performed in five replicates. Colony growth inhibition tests were performed by placing 6 mm mycelia agar disks, cut from the margin of expanding fungal colonies, in the centre of Petri dishes. The colony diameters were measured after different periods of incubation in the incubation chamber at 23 ºC in the dark. The first reading was taken when the colony diameter in control reached the border of Petri dish. The first readings were taken after two (*B. fuckeliana*), six (*P.digitatum*), seven (*M. laxa* and *M. fructigena*), nine (*Aspergillus* spp. and *P. expansum*) days of incubation because the hyphal growth and colony formation of fungi depend of fungal species.

### 3.8. Conidial Germination Test

Aliquots of plant extracts diluted in methanol were added to the autoclaved culture medium potato dextrose broth (PDB). The conidia concentration of each fungus 100 µL (6 × 10^5^ mL) with 900 µL of PDB (200 g of potato, 10 g of dextrose in 1 L distilled water) were mixed thoroughly to reach final concentration of 10, 50, 100, 200, 300 and 400 mg/L of plant extracts and a final concentration of conidia 6 × 10^4 ^mL. Final concentration of methanol didn’t exceed 0.3%. To increase the dispersion of extracts 0.1% of Tween 80 was added. Control in this test was PDB with 0.3% of methanol and 0.1% of Tween 80. To carry out the test, Petri dishes of 90 mm were used, a sterile filter paper was placed in each plate dripping 2 mL of sterile distilled water. A slide was fixed with the help of plastic supporter in the Petri dish. For each treatment two Petri dishes were prepared. In each slide two drops of 20 µL for each concentration were placed. After 10-12 hours at 23 ± 1 °C of incubation at dark conditions, the percentage of spore germination was determined microscopically at x40 magnification by counting 100 spores in each drop. Conidia were considered to have germinated when germ tube length was greater than the width of the conidium.

### 3.9. Statistical Analysis

One-way analysis of variance (ANOVA) of yielded extracts was used to determine the statistical significance; P-values< 0.01 were considered significant. The means were separated by Duncan’s multiple range test. The data were statistically analyzed using the software package STATISTICA 6, StatSoft Inc, Tulsa, USA. Also a principal component analysis (PCA) and cluster analysis was used to discriminate the main groups of extracts on the different fungi. The probit-analysis method was used to analyze the mean data of percentage inhibition of colony growth and conidia germination relative to concentration of each plant extracts. Percentage inhibition data were transformed to probit inhibition data. The regression lines of probit inhibition against log concentration were obtained and the effective concentration 50 (ED_50_) was calculated in mg/L according to linear model. Means were plotted against log_10_ values of the extract concentrations. MIC (minimum inhibitory concentration) was considered as the least concentration of plant extracts that does permit any visible growth of the inoculated mycelia or conidia during the incubation.

## 4. Conclusions

Results indicate that *D. viscose* possesses higher antifungal activity in comparison with that of *F. communis*, where information on its antifungal activity is limited or inexistent. *F. communis* root extract only affected colony growth of *B. fuckeliana, M. laxa* and *M. fructigena* and no inhibitory effects were observed on conidial germination under applied experimental concentrations. *F. communis* aerial part extract showed a lack of activity on colony growth and was not tested on conidia. 

As expected, *D. viscosa* extract demonstrated high activity on colony growth of most tested fungi, except *Aspergillus* spp. and *P. expansum*. In addition it posses the best effect on conidia germination of *M. laxa* and *M. fructigena* where data are still lacking. The extract of *D. viscosa* could be a potential alternative for the control of fungal rotting of fruits but further *in vivo* studies should be done.

Different degrees of antifungal activities can probably be explained by the varied chemical composition of the tested plant extracts. It seems that sesquiterpene lactones and acids from *D. viscosa* were more active than the daucane sesquitepenes and coumarin from *F. communis* against postharvest fungi. Antifungal activity can be associated with the compounds that occurred in extracts but the activities of pure compounds should be examined.

## Supplementary Materials

Supplementary File 1
