# Supplementary figures and images for: Chemical Composition and In Vitro Activity of Plant Extracts from Ferula communis and Dittrichia viscosa against Postharvest Fungi
Source: Molecules. 2011 Mar 22;16(3):2609–25. doi: 10.3390/molecules16032609 (PMC6259917; doi:10.3390/molecules16032609)

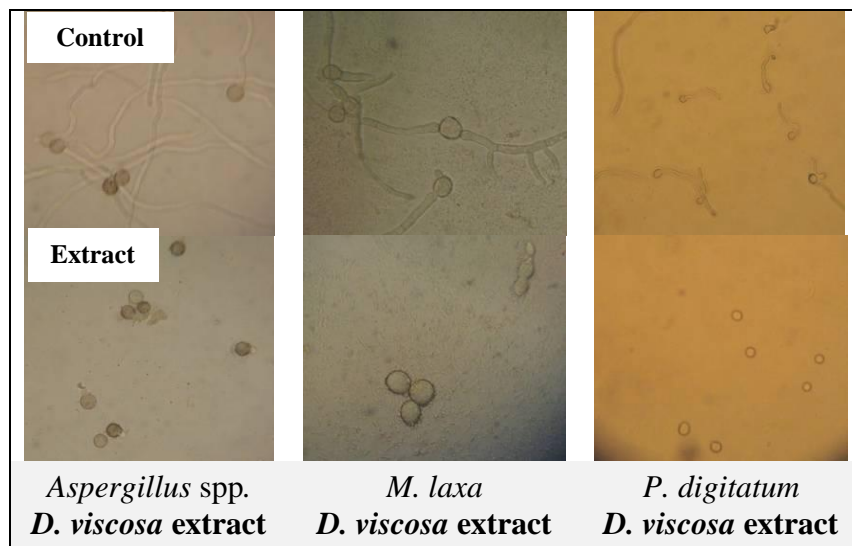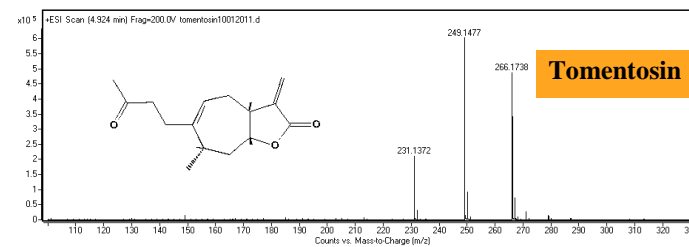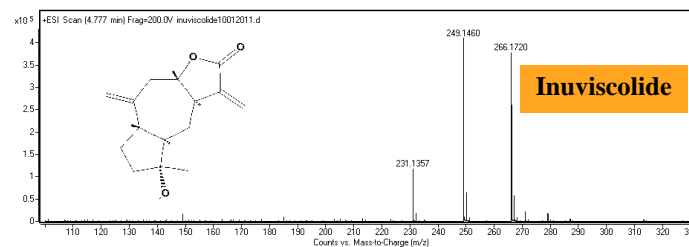

Supplement: Supplementary File 1 [file molecules-16-02609-s001.pdf]
